# Organic Acid Regulated Self-Assembly and Photophysical Properties of Perylene Bisimide Derivatives

**DOI:** 10.3390/ma13071656

**Published:** 2020-04-03

**Authors:** Ying Wang, Xinguo Yang, Siyu Li, Tao Long, Wei Li

**Affiliations:** College of Materials Science and Engineering, Hunan University, Changsha 410082, China; jhers950420@163.com (Y.W.); lisiyu199512@163.com (S.L.); longtao@hnu.edu.cn (T.L.); liwei5168@hnu.edu.cn (W.L.)

**Keywords:** perylene bisimide, self-assembly, organic acid, thermal sensitivity, gas sensitivity

## Abstract

In this work, perylene bisimide derivatives (PBI-1 and PBI-2) with tertiary amine groups were designed and synthesized. To control the final morphologies and properties of their aggregates, seven kinds of organic acids were used to alter the self-assembly environment. The influence of organic acids on the morphology of the aggregates was investigated. Photophysical properties of the aggregates were markedly affected by the kind and concentration of the organic acid. The thermal and gas sensitivities of the PBI-1 aggregates were studied with the use of UV–visible spectroscopy and digital imaging. The shift of the UV–visible spectra varied with time, temperature, acid type and acid concentration. Furthermore, PBI-1 aggregates showed a red-to-blue color change after addition of seven organic acids, whereas the color of the PBI-2 aggregates remained red. These changes of morphologies, photophysical properties and their thermal and gas sensitivities make these aggregates potentially useful in the fields of optoelectronics or sensors.

## 1. Introduction

In recent years, many functional nanomaterials have been synthesized through the use of self-assembly. The driving forces for self-assembly include non-covalent interaction forces, such as π–π interactions, hydrogen bonding, hydrophilic/hydrophobic interactions, van der Waals forces and charge interaction forces [1,2,3,4]. π-Conjugated systems have attracted great attention for applications to self-assembly mainly because of their designable molecular structures and excellent optoelectronic properties. However, the driving forces for self-assembly in π-conjugated systems involve π–π interactions, hydrogen bonding, van der Waals forces, and solvophobic interactions, and rarely include Coulomb forces. Coulomb forces interact with π–π interactions in self-assembly and can change the morphology and properties of aggregates. Specifically, Coulomb forces adjust the magnitude of π–π interactions and thus change the molecular stacking mode. This is an unusual case and is typically only achieved in fused ring molecules.

Perylene bisimide is an excellent π-conjugated system that has strong π–π stacking interactions and can form nanomaterials with various morphologies through ‘bottom-to-top’ self-assembly, such as rods, wires, belts and tubes [5,6,7,8]. These aggregate structures have a rich variety of optoelectronic properties; hence, perylene bisimide derivatives are widely used in solar cells [9,10,11,12], field effect transistors [13,14,15,16], light-emitting diodes [17,18,19] and sensors [20,21,22,23,24].

In general, the molecular structure and assembly conditions of perylene imide markedly affect the morphologies and properties of its aggregates. Zang [25] introduced linear dodecyl and branched nonyldecyl at the imide position of PBI, which aggregated into nanoribbons and into nanospheres when substituted with linear dodecyl chains and branched nonyldecyl chains, respectively. Yang [26] has reported that amphiphilic PBI molecules form nanotubes in methanol and nanoribbons in methylcyclohexane. Yao [27] synthesized PBI molecules by introducing a pyridine group at the bay position and discovered that adding hydrochloric acid changed the aggregates from nanodisks with weak fluorescence to nanospheres with strong fluorescence. Therefore, PBI aggregates with different morphologies, and optical properties can be obtained by changing the molecular structure, solvent polarity, pH value, molecular concentration, temperature and other conditions [28,29,30,31,32].

The tertiary amine group is a strong electron donor and can be easily protonated [33]. In our previous research, it was found that morphologies and properties of aggregates can be significantly influenced by hydrochloric acid and the molecular structure of PBI with tertiary amine groups. However, the situation of organic acids has not been involved, and there are few reports on the effect of organic acids on PBI aggregates.

In the present work, we report on the design and synthesis of PBI-1 and PBI-2 substituted with tertiary amine groups, and self-assembled in seven organic acids (namely, p-toluenesulfonic acid, oxalic acid, malonic acid, citric acid, formic acid, glutaric acid and acetic acid) through the use of the rapid solvent diffusion method. Morphologies, photophysical properties, and thermal and gas sensitivities of PBI-1 aggregates were investigated by digital imaging, polarized light microscopy (POM), field emission scanning electron microscope (SEM) imaging, UV–visible (UV–vis) spectroscopy and fluorescence spectroscopy. Changes of the morphologies and photophysical properties of PBI-1 aggregates assembled in organic acids are discussed. These results provide a basis for subsequent design of perylene bisimide nano-optical functional materials and their applications in sensors.

## 2. Experimental Section

### 2.1. Materials

All reagents were purchased from Shanghai Aladdin Bio-Chem Technology Co., Ltd. (Shanghai, China) and used directly without further purification. 1,6,7,12-Tetra(4-tert-butylphenoxy)-perylene-3,4,9,10-tetracarboxylic bisanhydride was prepared according to procedures described in the literature [34]; *N*,*N*-dimethylethylenediamine, 3-dimethylaminopropylamine and imidazole were of analytical reagent grade and used as received. Other reagents were analytically pure unless stated.

### 2.2. Synthesis and Characterization

#### 2.2.1. Synthesis of PBI-R

**Scheme 1:**
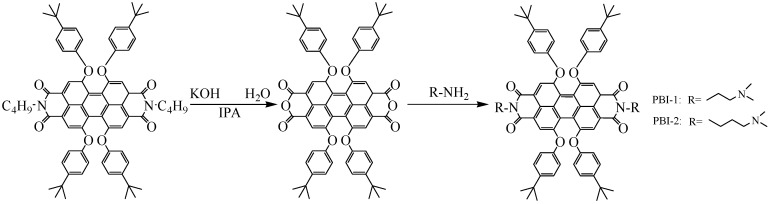
Synthetic route of PBI-R.

PBI-R was synthesized according to previously published procedures [35]. The synthesis route of PBI-R is shown in Scheme 1. The characterization data of R-PBI are shown in Figures S1–S6.

#### 2.2.2. Self-Assembly of PBI-R

**Scheme 2:**
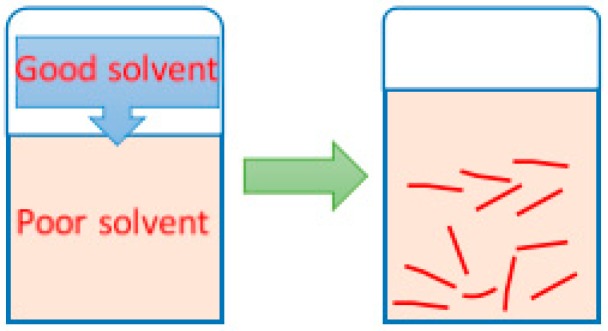
Self-assembly procedure of PBI-R.

The self-assembly of PBI-R was performed by a rapid solution diffusion method [36], as shown in Scheme 2. To ensure that the concentration of PBI-R aggregates was 3 × 10^−5^ mol/L, 1 mL of 1.8 × 10^−4^ mol/L of PBI-R tetrahydrofuran solution (good solvent) was rapidly injected into 5 mL of acid solution (poor solvent). This mixture was allowed to stand for 24 h until it completely mixed. The p-toluenesulfonic acid, oxalic acid, malonic acid, citric acid, formic acid, glutaric acid and acetic acid were used in the self-assembly experiments. The ratio of r [c(H^+^):c(the tertiary amine groups)] varied from 0:1 to 10,000:1 and 500:1 for PBI-1 and PBI-2, respectively (Table S1).

### 2.3. Characterization

Proton and carbon-13 nuclear magnetic resonance spectra of the samples were measured by INOVA-400 NMR analyzer (Varian, Inc, California, USA), and CDCl_3_ was used as solvent. FTIR spectra were recorded on a Nicolet-460 spectrometer (Thermo Fisher Scientific Inc, Massachusetts, USA) between 400 and 4000 cm^−1^ at room temperature by embedding the samples into KBr disks. The morphologies of the samples were characterized using a BX-51 polarized light microscope (Olympus Corporation, Tokyo, Japan). UV–vis absorption spectra were measured by a Shimadzu UV-2550 spectrometer (Shimadzu Corporation, Japan). Fluorescence spectra were recorded on a Hitachi F-2500 (Hitachi Corporation, Tokyo, Japan) spectrometer. X-ray diffraction (XRD) patterns were obtained on a Rigaku MiniFlex system with Cu-Ka radiation. The diffraction intensity was measured in a 2θ range between 5° and 90° at a scanning rate of 0.01°/s.

## 3. Results and Discussion

### 3.1. Chemical Structures Dependence

**Figure 1:**
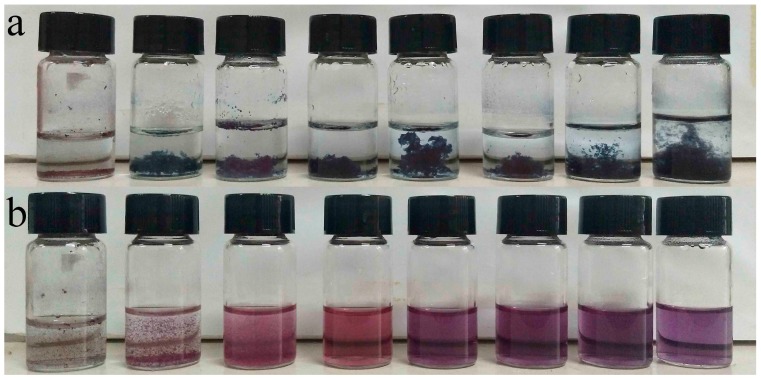
Digital images of PBI-R aggregates, r = 500:1, from left to right are water, p-toluenesulfonic acid, oxalic acid, malonic acid, citric acid, formic acid, glutaric acid and acetic acid. (**a**) PBI-1 and (**b**) PBI-2.

**Figure 2:**
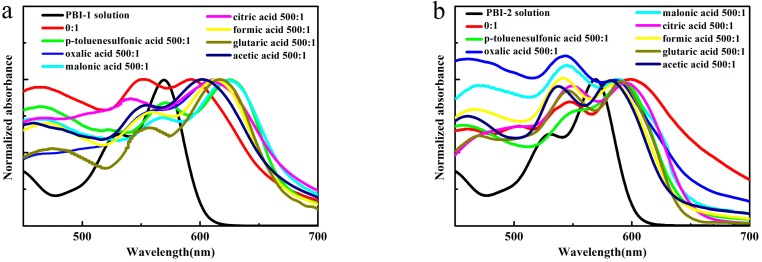
Normalized UV–visible (UV–vis) absorption spectra of PBI-R solution and PBI-R aggregates assembled in various organic acids. (**a**) PBI-1 and (**b**) PBI-2.

**Figure 3:**
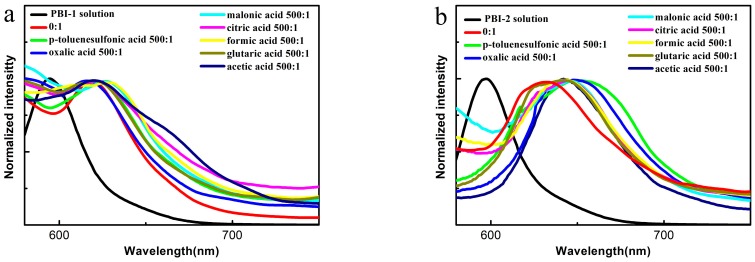
Normalized fluorescence emission spectrum of PBI-R solution and PBI-R aggregates assembled in various organic acids. (**a**) PBI-1 and (**b**) PBI-2.

In previous studies [35], we demonstrated that the molecular structure can affect aggregation in hydrochloric acid. However, the situation in organic acids was not discussed; hence, we used a digital camera and UV–vis absorption spectroscopy to study the PBI-R aggregates. Figure 1 indicates that the color of the PBI-1 aggregates changed from red to blue on addition of the seven organic acids, except for the PBI-2 aggregates, which remained red. As shown in UV–vis absorption spectra (Figure 2), the red shift of the PBI-2 aggregates was less than that of the PBI-1 aggregates. The value of A_0-0_/A_0-1_ (the ratio of the 0-0 to the 0-1 transition) is related to the degree of aggregation. When A_0-0_/A_0-1_ was close to 1.6, this indicated that the molecules existed in a single molecule state and that no aggregation occurred. The lower the value of A_0-0_/A_0-1_, the greater the degree of aggregation [30,37,38,39]. The A_0-0_/A_0-1_ of the PBI-R aggregates was calculated from the UV–vis absorption spectra; the A_0-0_/A_0-1_ of PBI-1 aggregates was approximately 1.2, whereas the A_0-0_/A_0-1_ of PBI-2 aggregates was approximately 0.9. From the fluorescence emission spectrum (Figure 3), we can see that the Stokes shift of the PBI-2 aggregates is about 60 nm compared with the UV–vis absorption spectra, which is almost twice that of the PBI-1 aggregates. These results suggest that two types of molecular stacking modes occurred in the PBI-1 and PBI-2 aggregates. The reason for this might be that the PBI-2 molecule has three carbon atoms between the tertiary amine group and the core, and the protonated tertiary amine groups are far from the core plane. The charge repulsion interaction has a slight effect on PBI-2 molecular aggregation; hence, the intermolecular π–π interactions of the PBI-2 molecule are stronger. This study confirms that chemical structures have an important influence over the aggregation behavior in organic acids. Here, we will discuss the properties of PBI-1 aggregates assembled in various organic acids.

### 3.2. Morphologies of PBI-1 Aggregates

**Figure 4:**
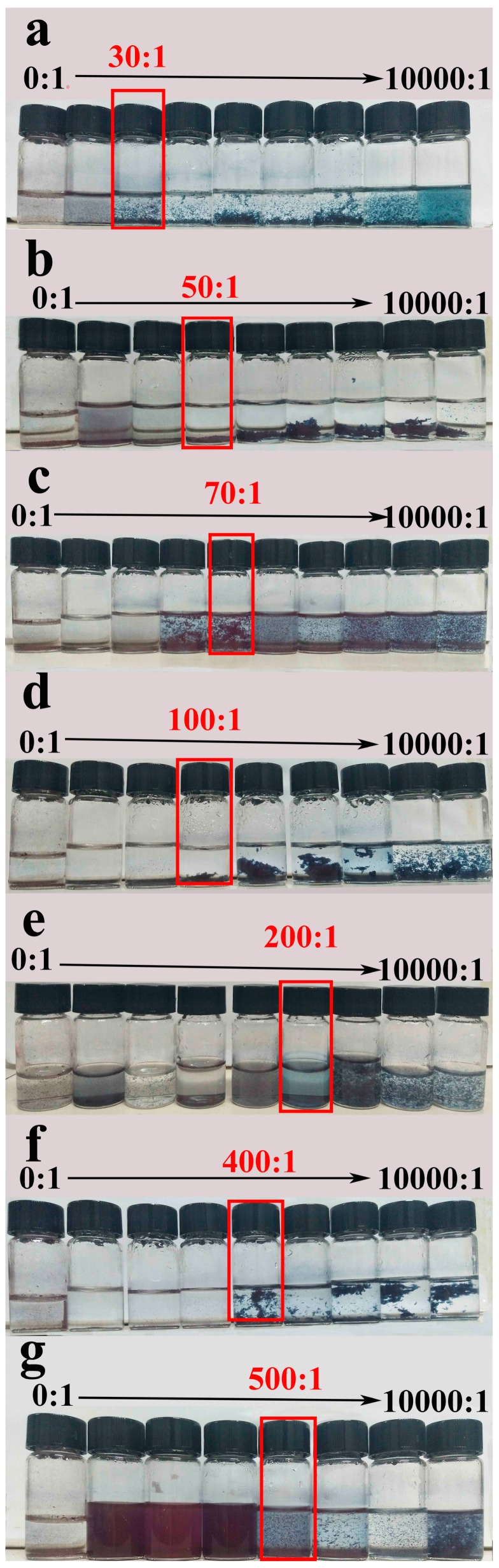
Digital image of PBI-1 aggregates assembled in various organic acids corresponding to different r values, from 0:1 to 10,000:1. (**a**) p-Toluenesulfonic acid, (**b**) oxalic acid, (**c**) malonic acid, (**d**) citric acid, (**e**) formic acid, (**f**) glutaric acid and (**g**) acetic acid.

Figure 4 shows a digital image of the PBI-1 aggregates assembled in seven organic acids. The specific concentration of the organic acid changed the color of PBI-1 aggregates, that is, at r = 30:1 for p-toluenesulfonic acid, 50:1 for oxalic acid, 70:1 for malonic acid, 100:1 for citric acid, 200:1 for formic acid, 400:1 for glutaric acid and 500:1 for acetic acid. As shown in Table 1, p-toluenesulfonic acid is a strong organic acid with the lowest pKa among these seven organic acids. Hence, this acid is the strongest protonation agent and gives a sharp color change at a low r value. We conclude that the pKa of the organic acids determines the r value of the color transition.

**Figure 5:**
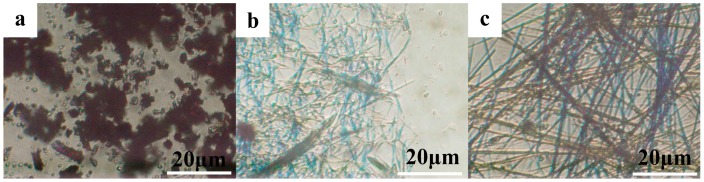
Optical microscope images of PBI-1 aggregates assembled in p-toluenesulfonic acid. (**a**) r = 0:1, (**b**) r = 50:1 and (**c**) r = 10,000:1.

**Figure 6:**
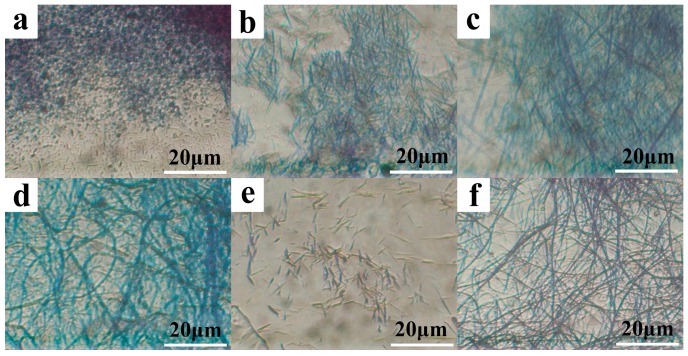
Optical microscope images of PBI-1 aggregates assembled in various organic acids. (**a**) r = 10,000:1 oxalic acid, (**b**) r = 5000:1 malonic acid, (**c**) r = 10,000:1 citric acid, (**d**) r = 10,000:1 formic acid, (**e**) r = 10,000:1 glutaric acid and (**f**) r = 5000:1 acetic acid.

**Figure 7:**
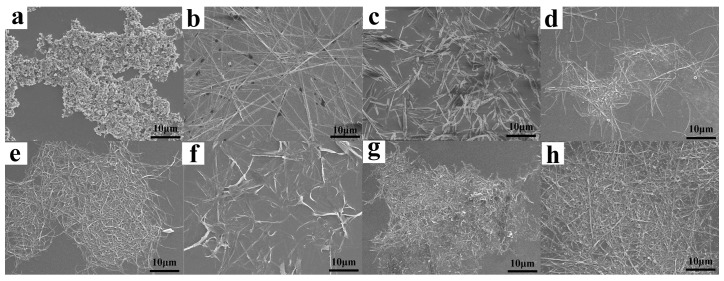
Scanning electron microscopy (SEM) images of PBI-1 aggregates assembled in various organic acids, r = 5000:1. (**a**) No added acid, (**b**) p-toluenesulfonic acid, (**c**) oxalic acid, (**d**) malonic acid, (**e**) citric acid, (**f**) formic acid, (**g**) glutaric acid and (**h**) acetic acid.

Morphologies of PBI-1 aggregates assembled in various organic acids were observed in detail with the use of POM (Figure 5 and Figure 6) and SEM (Figure 7). As shown in Figure 5, when r = 0:1, that is, without organic acid, PBI-1 assembled into massive irregular aggregates. After p-toluenesulfonic acid was added, the fine fibers began to grow. The number of fibers increased, and they lengthened as the r value increased. The same results (Figure S7) were observed in oxalic acid, malonic acid, citric acid, formic acid, glutaric acid and acetic acid. It is likely that in the presence of only water, the driving forces of self-assembly are π–π stacking and hydrophobic interaction; hence, PBI-1 molecules will assemble quickly by hydrophobic interactions. At this time, π–π stacking is too slow to make PBI-1 molecules completely align in a certain direction, which results in irregular aggregates [34]. After the organic acid was added, the organic acid ionized to generate H^+^, protonating the tertiary amine. The protonated tertiary amine groups induce charge repulsion interactions and π–π stacking interactions on PBI-1 molecules in addition to hydrophobic interactions. The degree of protonation of the tertiary amine groups increased as r increased, which is more conducive to an ordered arrangement; hence, the fibers lengthened.

**Figure 8:**
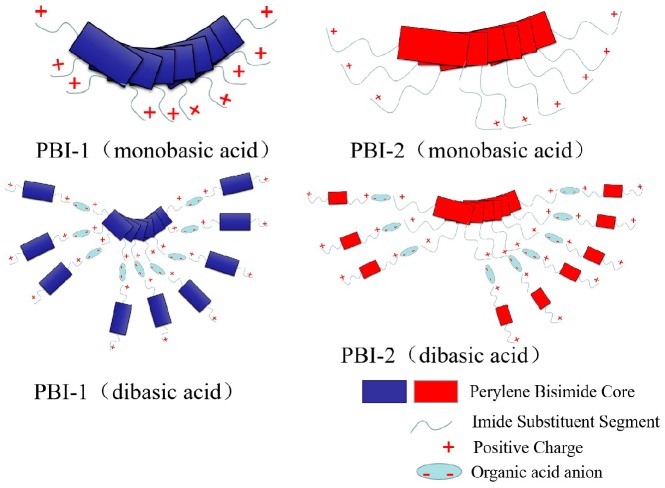
Proposed schematic diagrams of self-assembly for PBI-1 and PBI-2.

As shown in Figure 6 and Figure 7, fibers in dibasic and tribasic acids were shorter than those in monobasic acids. The molecular structure of the organic acid anions also affected the self-assembly process. Oxalic acid, malonic acid and glutaric acid are dibasic acids; one diacid molecule might be connected to two PBI-1 molecules during ionization, which limits the ordered arrangement of molecules (Figure 8); hence, only short fibers can be formed. Similarly, tribasic acid and citric acid can only form short fibers. In addition, there are some changes in the molecular stacking direction owing to charge repulsion interactions, which induce the formation of curved fibers.

From the above results, we concluded that both the degree of protonation of the tertiary amine group and molecular structures of organic acids have considerable effects on the structure of PBI-1 aggregates, which is because the kinds and concentrations of organic acids lead to different driving forces in the self-assembly process.

### 3.3. Photophysical Properties of PBI-1 Aggregates

**Figure 9:**
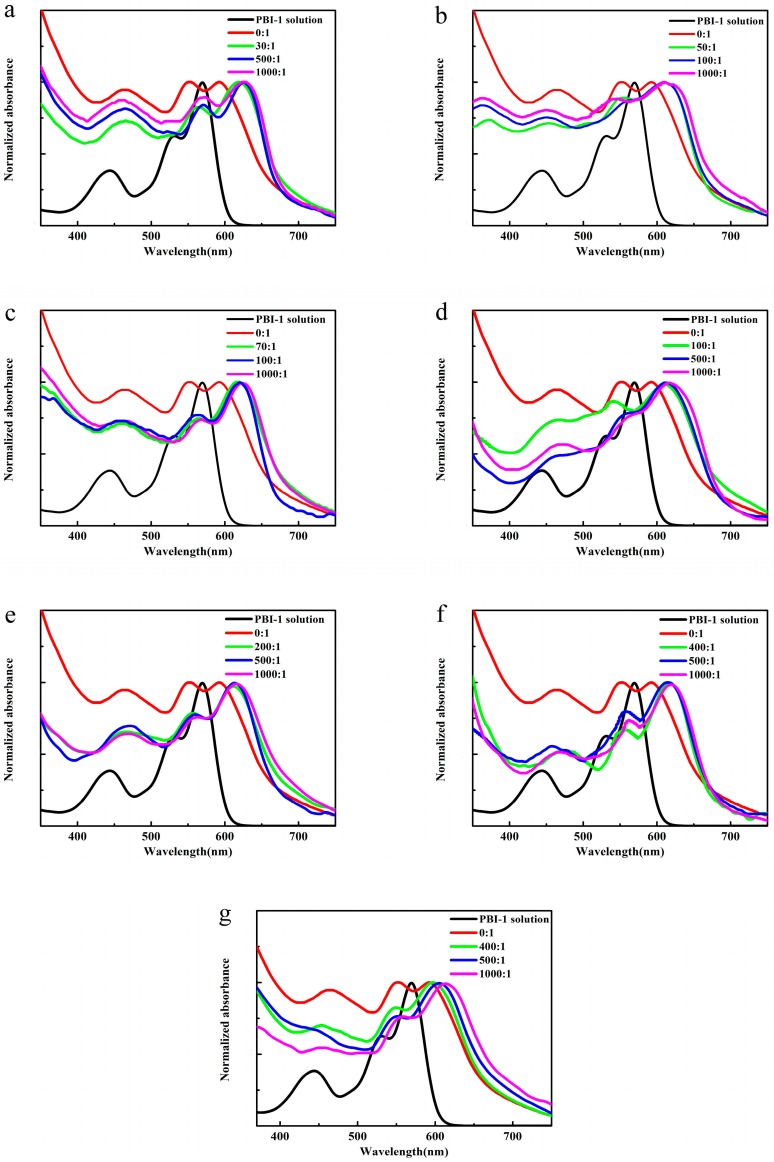
Normalized UV–vis absorption spectra of PBI-1 solution and PBI-1 aggregates at different r values assembled in various organic acids. (**a**) p-Toluenesulfonic acid, (**b**) oxalic acid, (**c**) malonic acid, (**d**) citric acid, (**e**) formic acid, (**f**) glutaric acid and (**g**) acetic acid.

To further explore the effects of organic acids on the photophysical properties of PBI-1 aggregates, we performed UV–vis absorption spectroscopy and fluorescence emission spectroscopy of PBI-1 aggregates. Figure 9 shows the normalized UV–vis absorption spectrum of PBI-1 solution and a part of the PBI-1 aggregates in different assembly conditions. The UV–vis absorption spectrum of PBI-1 in THF solution had three absorption peaks at 440, 530 and 570 nm, which represent 0-2, 0-1 and 0-0 electron transitions, respectively [25,40,41,42]. When r = 0:1, the maximum UV–vis absorption peak of the PBI-1 aggregates red-shifted from 570 to 590 nm compared with PBI-1 solution. After adding organic acid, the red shift increased. For the same organic acid, the red shift increased as the r value increased. When r increased from 0:1 to 1000:1, the red shift of PBI-1 aggregates assembled in p-toluenesulfonic acid (Figure 9a) was the largest, which is approximately 40 nm compared with that of no organic acid. The red shifts of the PBI-1 aggregates assembled in oxalic acid, malonic acid and citric acid were approximately 35 nm (Figure 9b–d), that in formic acid was approximately 30 nm (Figure 9e) and those in glutaric acid and acetic acid were approximately 25 nm (Figure 9f,g). Additionally, after adding the organic acid, the A_0-0_/A_0-1_ value was markedly lowered. The values of A_0-0_/A_0-1_ for p-toluenesulfonic acid, oxalic acid, malonic acid, citric acid, formic acid, glutaric acid and acetic acid were approximately 1.19, 1.21, 1.26, 1.29, 1.31, 1.32 and 1.32, respectively (Table S2). The UV–vis spectra indicated that the π–π interaction was greatest in acetic acid owing to its minimal red shift [43,44]. The molecular stacking mode of the aggregates was J-type aggregation. This is because charge repulsion interactions increase as r increases and the pKa decreases; hence, π–π interactions decrease. The PBI-1 molecular bay positions are replaced by tert-butylphenoxy groups, which have a large steric hindrance that destroys the planarity of the PBI molecules; hence, PBI-1 molecules aggregate in a J-type manner when stacked [45,46]. The above results indicate that the degree of protonation has notable effects on the photophysical properties of PBI-1 aggregates.

**Figure 10:**
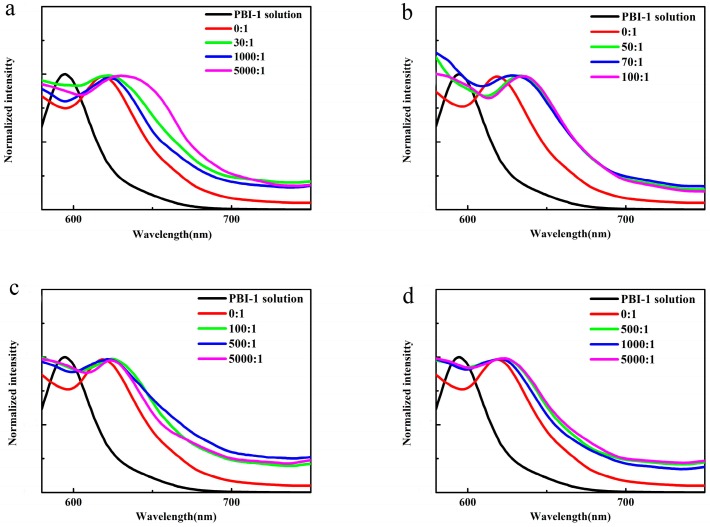
Normalized fluorescence emission spectra of PBI-1 solution and PBI-1 aggregates at different r values assembled in various organic acids. (**a**) p-Toluenesulfonic acid, (**b**) oxalic acid, (**c**) citric acid and (**d**) glutaric acid. λ_ex_ = 440 nm was used to record the emission spectra.

Figure 10 shows the normalized fluorescence emission spectrum of PBI-1 solution and a part of the PBI-1 aggregated under the different assembly conditions. The fluorescence emission spectrum of PBI-1 solution had an emission peak at approximately 595 nm and a Stokes shift of approximately 25 nm. The fluorescence emission spectrum of the PBI-1 aggregates assembled in organic acid solution had a maximum emission peak at 620–640 nm. Additionally, the bathochromic shift of the emission peak increased as r increased and the pK_a_ decreased, which is consistent with the results of the UV–vis absorption spectra. The formation of J-aggregates can be further confirmed by a bathochromic shift of fluorescence emission peak [27,47]. The fluorescence of the PBI-1 aggregates was not quenched, which might be attributable to the charge repulsion interaction provided by protonated tertiary amine groups, which weakened the molecular π–π interactions; hence, the aggregates retained their fluorescence.

### 3.4. Molecular Stacking of PBI-1 Aggregates

**Figure 11:**
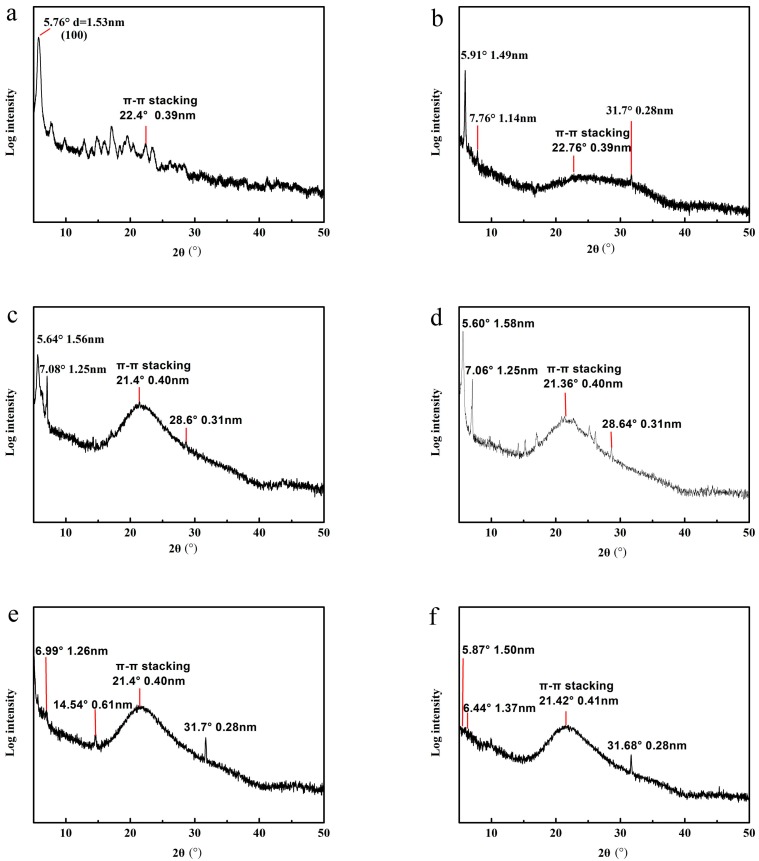
X-ray diffraction (XRD) of PBI-1 powder and partial aggregates. (**a**) PBI-1 powder, (**b**) r = 0:1, (**c**) r = 30:1 p-toluenesulfonic acid, (**d**) r = 100:1 p-toluenesulfonic acid, (**e**) r = 200:1 formic acid and (**f**) r = 500:1 acetic acid.

To further study the internal structure of PBI-1 aggregates, XRD was performed on PBI-1 powder and partial aggregates (Figure 11). As can be seen from Figure 11a, the PBI-1 powder had a diffraction peak at 2θ = 5.76° (1.53 nm), which corresponds to the (100) plane and can be assigned to the lengths of the PBI-1 units [8]. The low-intensity diffraction peak appearing at 2θ = 22.4° (0.39 nm) is attributed to π–π stacking of adjacent PBI-1 molecules, which indicates that the π–π stacking effect is weak [48,49]. The diffraction peak of PBI-1 aggregates at 22.76° (0.39 nm) broadens to form a taro peak when r = 0:1 (Figure 11b), which indicates that the π–π stacking effect strengthens [48,50]. In addition to the π–π stacking peak of 0.40 nm, the d values of the three diffraction peaks become greater for the p-toluenesulfonic acid system, and the d value increased from 1.56 to 1.58 nm when r increased from 30:1 to 100:1 (Figure 11c,d); whereas the diffraction peak was basically the same as r = 0:1 in the wide-angle region for formic acid and acetic acid system, and the d value of the diffraction peak in the small angle region decreased (Figure 11e,f). Hence, there are some notable differences in the self-assembly process of PBI-1 molecules by XRD. This result is consistent with information obtained from the UV–vis absorption spectra.

### 3.5. Thermal Sensitivity of PBI-1 Aggregates

**Figure 12:**
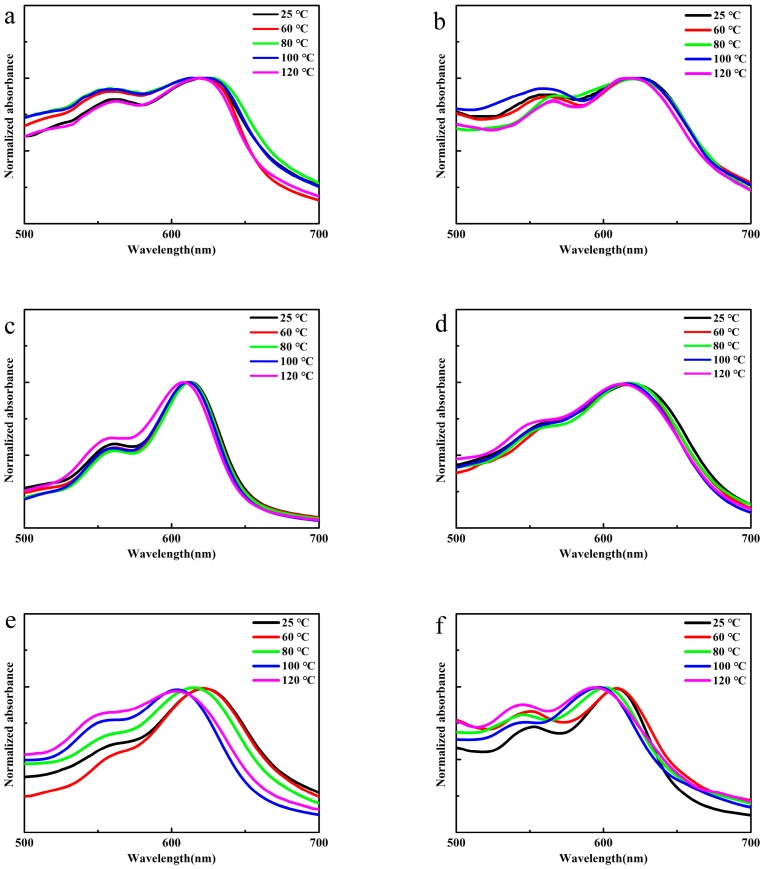
Normalized UV–vis absorption spectra of PBI-1 aggregates after 2 h heating. (**a**) r = 15:1 hydrochloric acid, (**b**) r = 30:1 p-toluenesulfonic acid, (**c**) r = 100:1 malonic acid, (**d**) r = 200:1 citric acid, (**e**) r = 500:1 glutaric acid and (**f**) r = 500:1 adipic acid.

**Figure 13:**
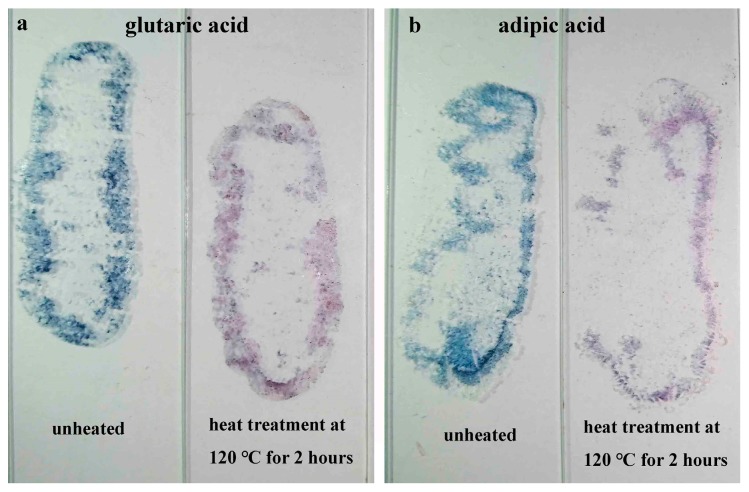
Digital image of PBI-1 aggregates, r = 500:1.

To investigate the thermal sensitivity of PBI-1 aggregates, UV–vis absorption spectra of the heated aggregates were measured. As shown in Figure 12, when the temperature of aggregates was gradually increased to 120 °C and held for 2 h, the UV–vis absorption spectra did not change in hydrochloric acid (Figure 12a), p-toluenesulfonic acid (Figure 12b), formic acid (Figure S8b) or acetic acid (Figure S8c); the UV–vis absorption spectra blue-shifted in oxalic acid (Figure S8a), malonic acid (Figure 12c), glutaric acid (Figure 12e) and citric acid (Figure 12d) by approximately 7, 10, 20 and 10 nm, respectively. The color of the aggregates formed in glutaric acid changed from blue to red after heating at 120 °C for 2 h (Figure 13a). At the same time, we also confirmed that the same phenomenon exists in the aggregates formed in the adipic acid (Figure S9, Figure 12f and Figure 13b). These results indicate that PBI-1 aggregates formed in dibasic and tribasic acids are sensitive to temperature.

Reasonable explanations for these observations are that charge repulsion interactions and the large steric hindrance reduced the overlapping area of aromatic rings of adjacent PBI-1 molecules in dibasic and tribasic acids, such that π–π interactions were weaker than those in monobasic acids. Therefore, UV–vis absorption spectra of aggregates formed in dibasic and tribasic acids blue-shifted after heating. Among these, glutaric acid has a relatively flexible alkyl chain, which is conducive to molecule movement. Therefore, aggregates formed in glutaric acid had the maximum blue shift after heating.

### 3.6. Gas Sensitivity of PBI-1 Aggregates

**Figure 14:**
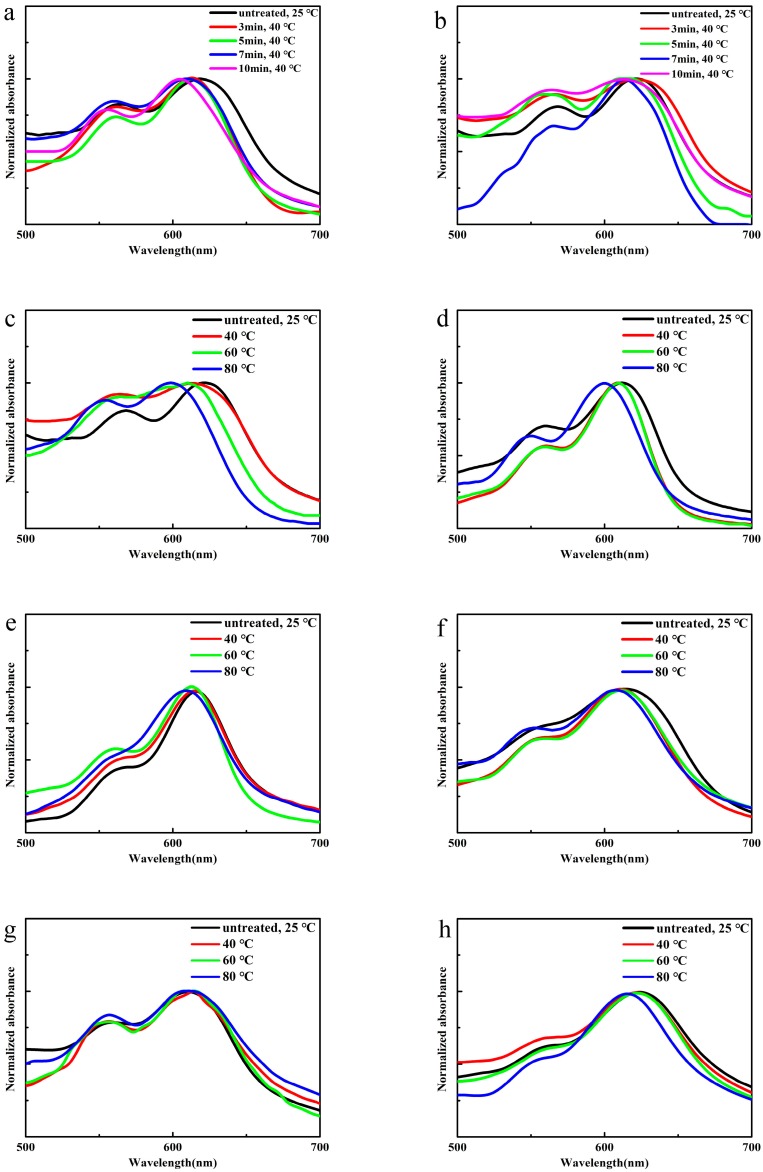
Normalized UV–vis absorption spectra of PBI-1 aggregates with ammonia treatment. (**a**) r = 30:1 p-toluenesulfonic acid, 40 °C, (**b**) r = 50:1 p-toluenesulfonic acid, 40 °C, (**c**) r = 50:1 p-toluenesulfonic acid, 10 min, (**d**) r = 50:1 oxalic acid, 10 min, (**e**) r = 100:1 malonic acid, 10 min, (**f**) r = 200:1 citric acid, 10 min, (**g**) r = 500:1 formic acid, 10 min and (**h**) r = 500:1 glutaric acid, 10 min.

To investigate the gas sensitivity of PBI-1 aggregates, ammonia-treated aggregates were characterized using UV–vis absorption spectroscopy. As shown in Figure 14a,b, UV–vis absorption spectra of the PBI-1 aggregates blue-shifted after the ammonia treatment at the same r value, and the blue shift gradually increased as treatment time increased. In the p-toluenesulfonic acid system, the blue shift became approximately 15 nm as the treatment time was increased from 0 to 10 min. Figure 14c–h indicates that the blue shift gradually increased as the treatment temperature increased for the same ammonia treatment time. For example, the UV–vis absorption spectrum blue-shifted by 25 nm when the temperature was raised to 80 °C for the p-toluenesulfonic acid system; for the oxalic acid system, the blue shift was approximately 15 nm; for the malonic acid, citric acid and glutaric acid systems, the blue shifts were approximately 7 nm. The UV–vis absorption spectrum did not change in the formic acid system.

This phenomenon might be attributable to ammonia gas reacting with protonated tertiary amine groups, which changes the aggregation of PBI-1 aggregates and the maximum absorption blue shift. The reaction proceeded to completion as the temperature was increased and the time prolonged, which led to a greater blue shift.

## 4. Conclusions

In summary, we designed and synthesized PBI-1 and PBI-2 molecules with tertiary amine substituents. The color of the PBI-1 aggregates changed from red to blue after addition of seven organic acids, and the color of PBI-2 aggregates remained red. We have demonstrated that protonation of tertiary amine groups and the anionic structure of organic acids affect the aggregation of PBI-1. The charge repulsion interactions provided by organic acids synergistically interact with other non-covalent bonding forces to transform irregular PBI-1 aggregates into well-defined nanofibers. The preparation method is simple, and morphologies, colors and photophysical properties are controllable. Additionally, the system has a certain sensitivity to temperature and ammonia, which suggests the potential for application of PBI-1 aggregates in optoelectronic devices or sensors.

## Figures and Tables

**Table 1:** pKa values of several organic acids.

	p-Toluenesulfonic Acid	Oxalic Acid	Malonic Acid	Citric Acid	Formic Acid	Glutaric Acid	Acetic Acid	Adipic Acid
pKa	−0.43 ± 0.50	1.23	2.83	3.14	3.75	4.31	4.74	4.43

## Supplementary Materials

The following are available online at https://www.mdpi.com/1996-1944/13/7/1656/s1, Figure S1: 1H NMR spectrum of PBI-1 in CDCl3, Figure S2: 13C NMR spectrum of PBI-1 in CDCl3, Figure S3: FT-IR spectrum of PBI-1, Figure S4: 1H NMR spectrum of PBI-2 in CDCl3, Figure S5: 13C NMR spectrum of PBI-2 in CDCl3, Figure S6: FT-IR spectrum of PBI-2, Figure S7: Optical microscope images of PBI-1 aggregates at different r assembled in various organic acids, Figure S8: Normalized UV-vis absorption spectra of PBI-1 aggregates after 2 h heating. (**a**) r = 50:1 oxalic acid, (**b**) r = 300:1 formic acid, (**c**) r = 500:1 acetic acid, Figure S9: Digital image of PBI-1 aggregates assembled in adipic acid, r = 500:1, Table S1: Organic acid concentrations correspond to different r parameters, Table S2: The value of A_0-0_/A_0-1_ of PBI-1 aggregates at different r assembled in various organic acids.
