# Supplementary material for: Organic Acid Regulated Self-Assembly and Photophysical Properties of Perylene Bisimide Derivatives
Source: Materials (Basel). 2020 Apr 3;13(7):1656. doi: 10.3390/ma13071656 (PMC7178315; doi:10.3390/ma13071656)
Supplement: Supplementary file 1 [file materials-13-01656-s001.pdf]

Supplementary Materials

Organic Acid Regulated Self-Assembly and Photophysical Properties of Perylene Bisimide Derivatives

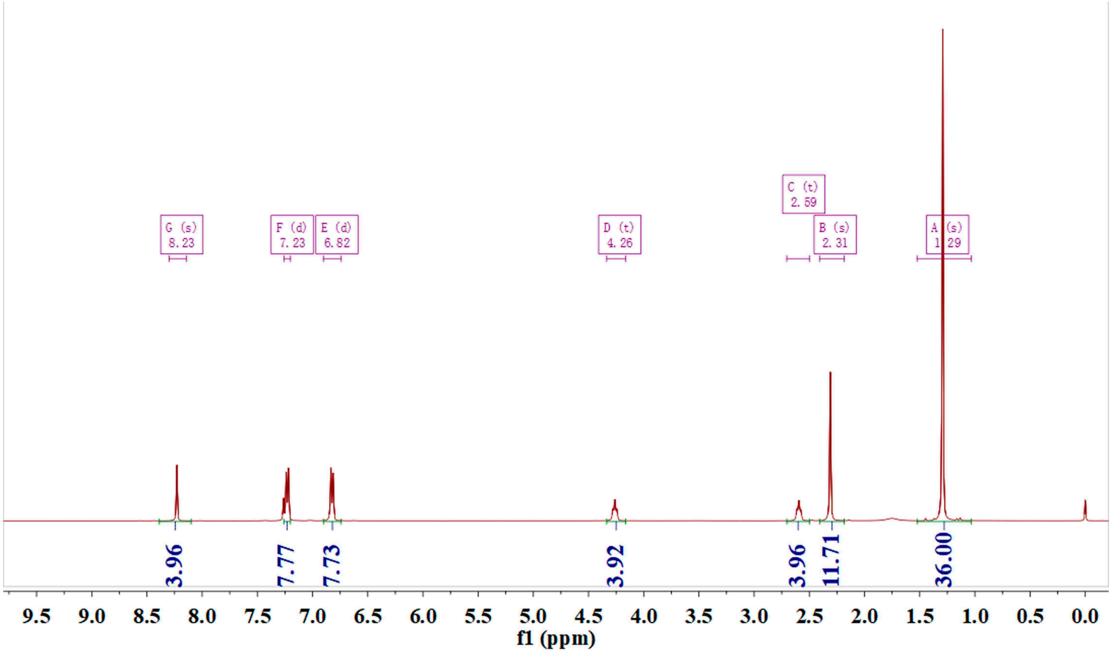

Figure S1. 1H NMR spectrum of PBI-1 in CDCl3.

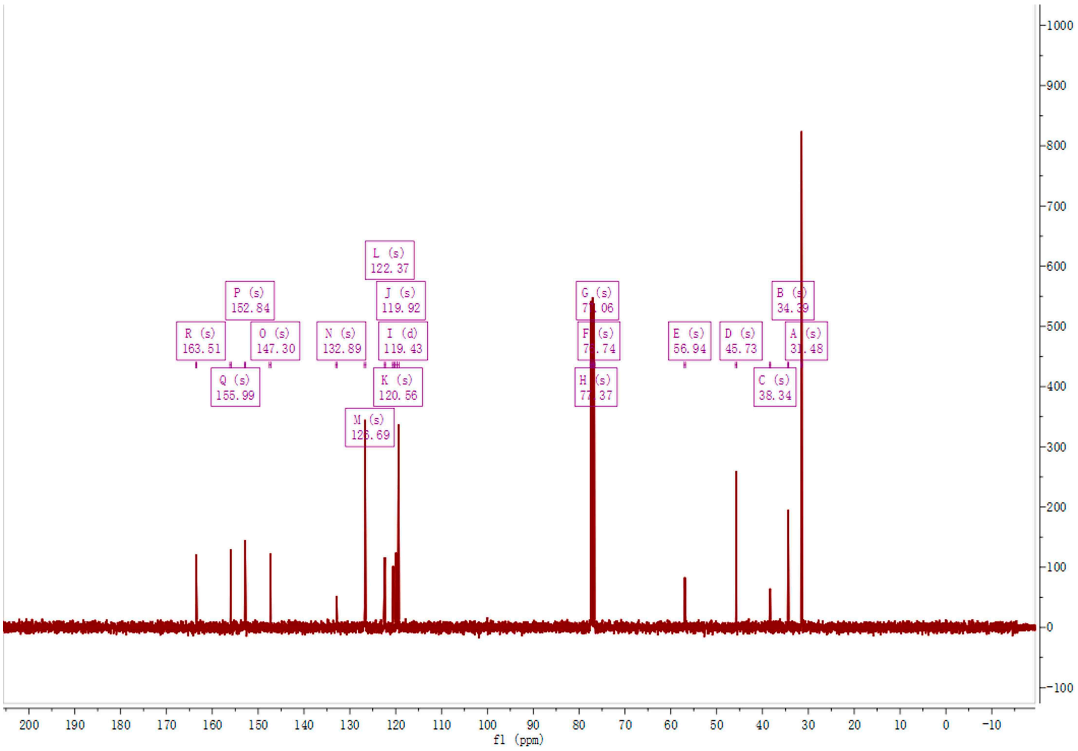

Figure S2. 13C NMR spectrum of PBI-1 in CDCl3.

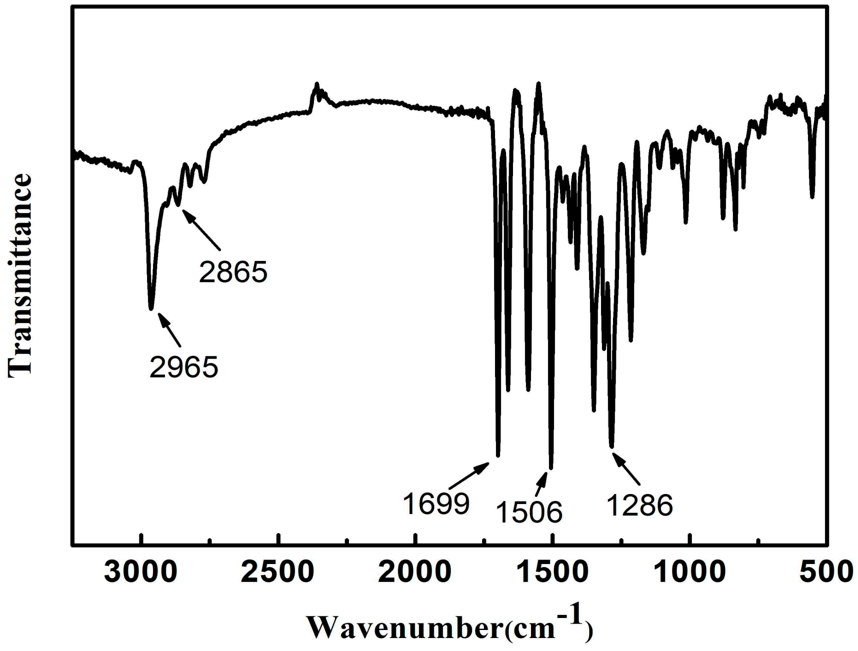

Figure S3. FT-IR spectrum of PBI-1.

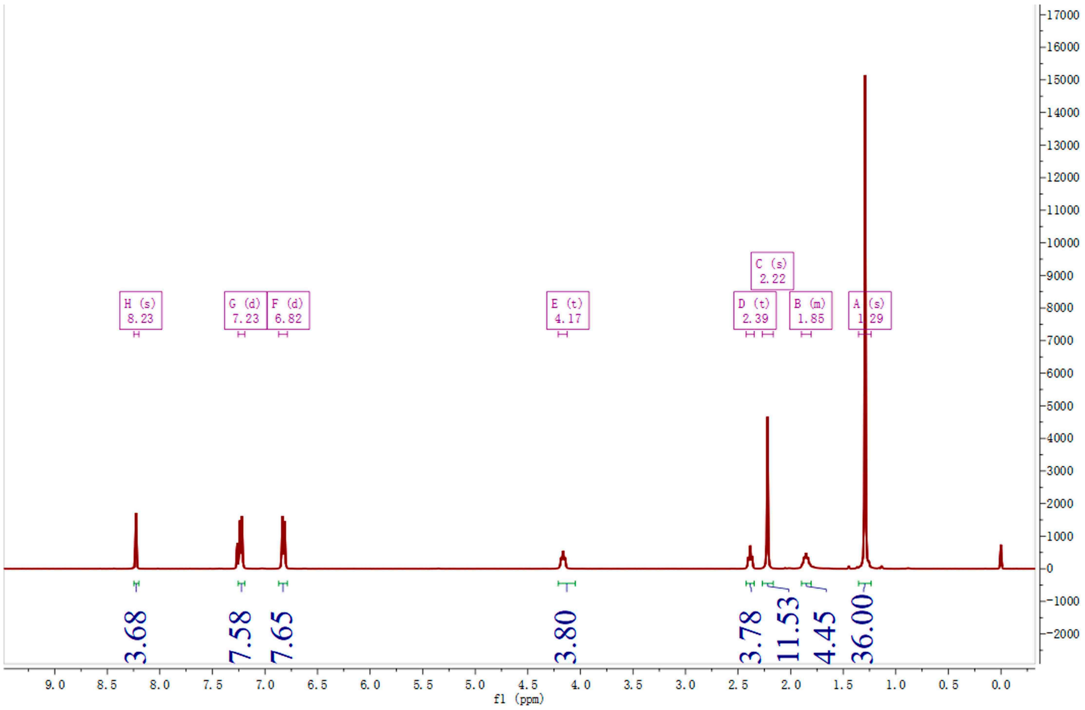

Figure S4. 1H NMR spectrum of PBI-2 in CDCl3.

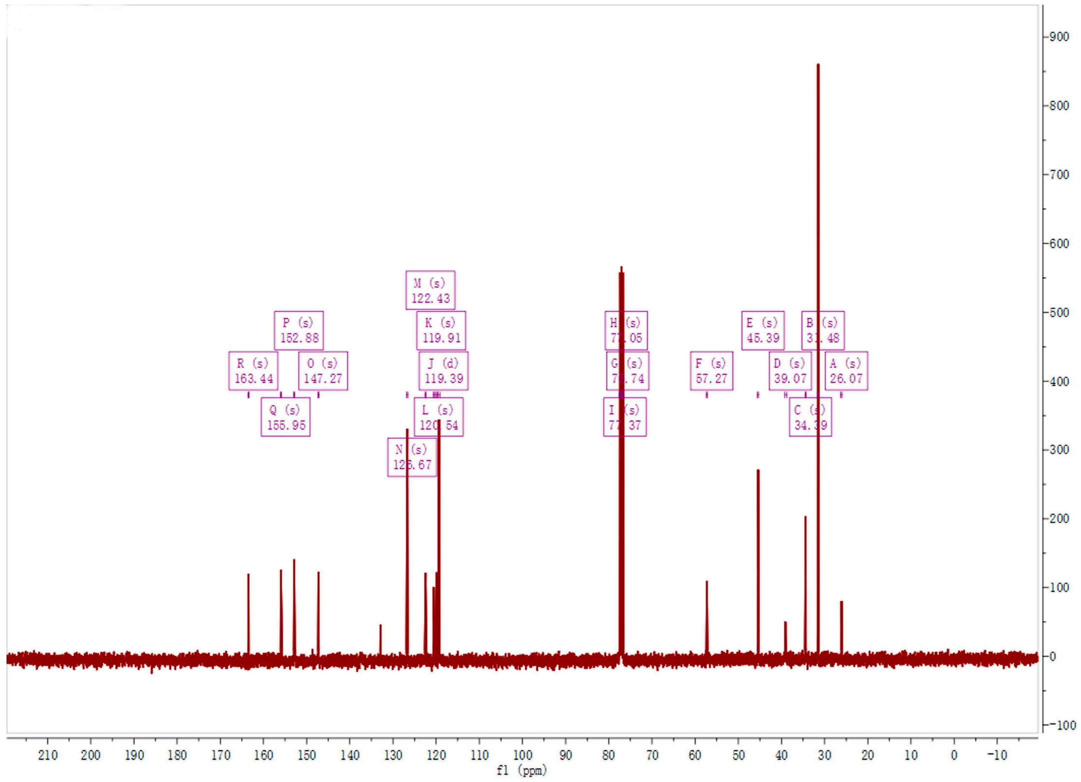

Figure S5. 13C NMR spectrum of PBI-2 in CDCl3.

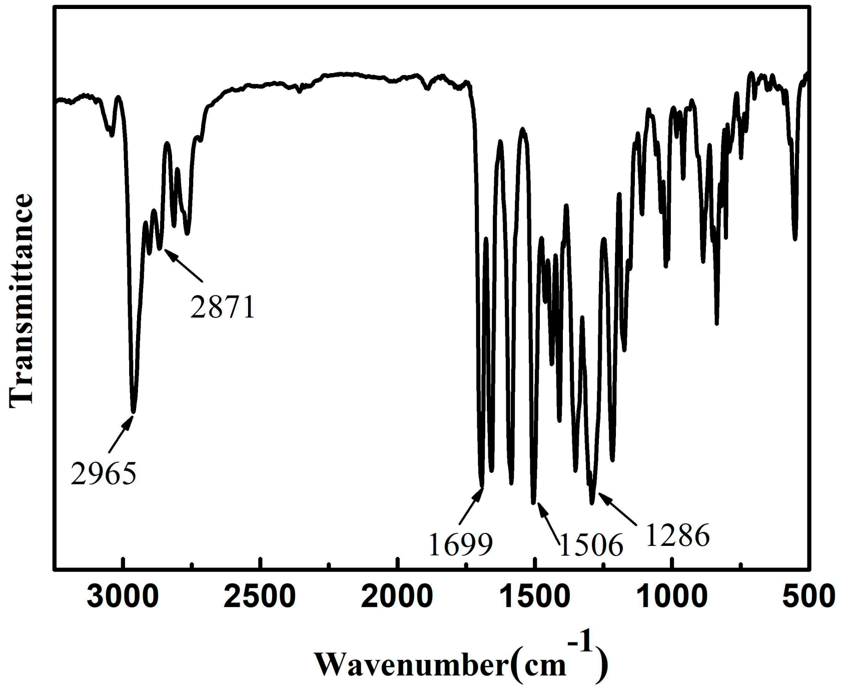

Figure S6. FT-IR spectrum of PBI-2.

Table S1. Organic acid concentrations correspond to different r parameters.

**r**	**c(C7H8O3S)/mol/L**	**c(C2H2O4)/mol/L**	**c(C3H4O4)/mol/L**	**c(C6H8O7)/mol/L**	**c(CH2O2)/mol/L**	**c(C5H8O4)/mol/L**	**c(C2H4O2)/mol/L**
0:1	0	0	0	0	0	0	0
10:1	7.2×10^{-3}	3.6×10^{-3}	3.6×10^{-3}	2.4×10^{-3}	7.2×10^{-3}	3.6×10^{-3}	7.2×10^{-3}
30:1	2.16×10^{-2}	1.08×10^{-2}	1.08×10^{-2}	7.2×10^{-3}	2.16×10^{-2}	1.08×10^{-2}	2.16×10^{-2}
50:1	3.6×10^{-2}	1.8×10^{-2}	1.8×10^{-2}	1.2×10^{-2}	3.6×10^{-2}	1.8×10^{-2}	3.6×10^{-2}
70:1	5.04×10^{-2}	2.52×10^{-2}	2.52×10^{-2}	1.68×10^{-2}	5.04×10^{-2}	2.52×10^{-2}	5.04×10^{-2}
100:1	7.2×10^{-2}	3.6×10^{-2}	3.6×10^{-2}	2.4×10^{-2}	7.2×10^{-2}	3.6×10^{-2}	7.2×10^{-2}
200:1	1.44×10^{-2}	7.2×10^{-2}	7.2×10^{-2}	4.8×10^{-2}	1.44×10^{-2}	7.2×10^{-2}	1.44×10^{-2}
300:1	0.216	0.108	0.108	0.072	0.216	0.108	0.216
500:1	0.36	0.18	0.18	0.12	0.36	0.18	0.36
1000:1	0.72	0.36	0.36	0.24	0.72	0.36	0.72
3000:1	2.16	1.08	1.08	0.72	2.16	1.08	2.16
5000:1	3.6	1.8	1.8	1.2	3.6	1.8	3.6
10000:1	7.2	3.6	3.6	2.4	7.2	3.6	7.2

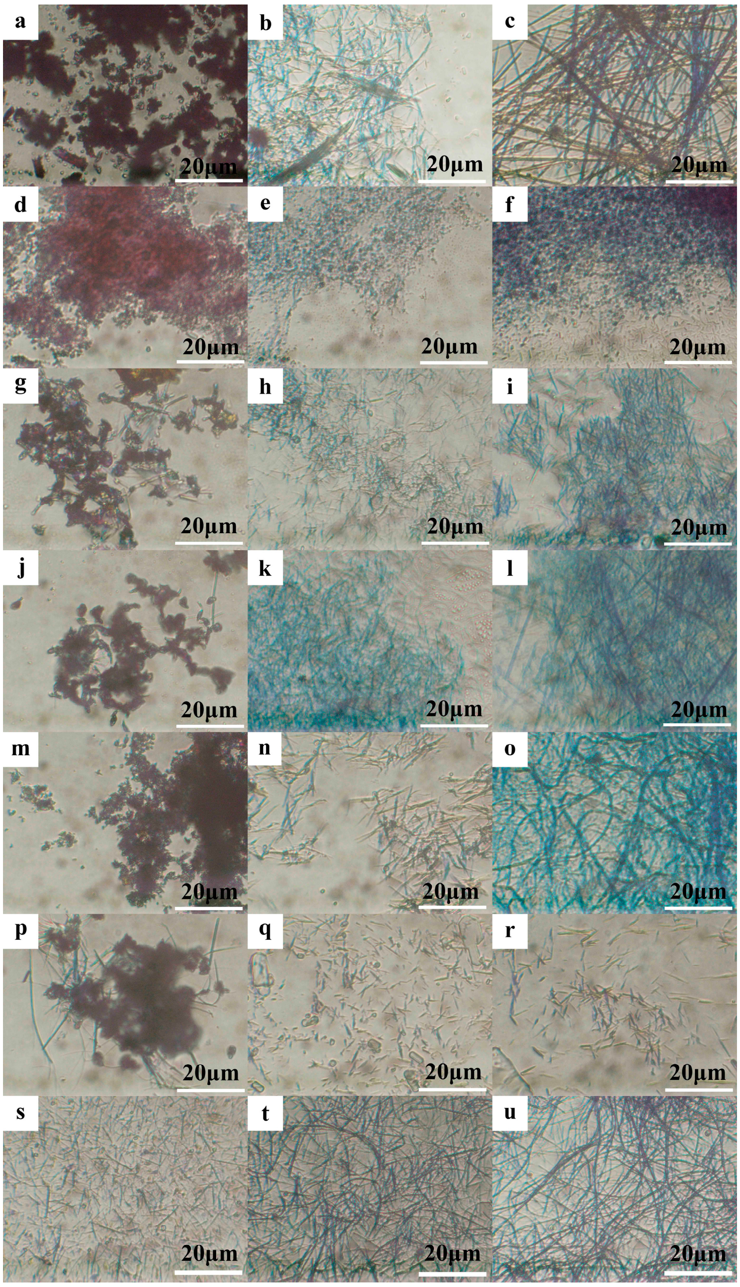

Figure S7. Optical microscope images of PBI-1 aggregates at different r assembled in various organic acids.

(**a**) r = 0:1, p-toluenesulfonic acid: (**b**) r = 50:1, (**c**) r = 10000:1,

oxalic acid: (**d**) r = 10:1, (**e**) r = 70:1, (**f**) r = 10000:1,

malonic acid: (**g**) r = 50:1, (**h**) r = 100:1, (**i**) r = 5000:1,

citric acid: (**j**) r = 50:1, (**k**) r = 500:1, (**l**) r = 10000:1,

formic acid: (**m**) r = 50:1, (**n**) r = 300:1, (**o**) r = 10000:1,

glutaric acid: (**p**) r = 50:1, (**q**) r = 500:1, (**r**) r = 10000:1,

acetic acid: (**s**) r = 500:1, (**t**) r = 1000:1, (**u**) r = 5000:1.

Table S2. The value of A_{0-0}/A_{0-1} of PBI-1 aggregates at different r assembled in various organic acids.

A_{0-0}/A_{0-1}	30:1	50:1	70:1	100:1	200:1	400:1	500:1	1000:1	Average Value
p-toluenesulf onic acid	1.22	1.22	1.16	1.18	1.21	1.20	1.20	1.14	1.19
oxalic acid	1.26	1.17	1.19	1.24	1.23	1.19	1.24	1.16	1.21
malonic acid	1.22	1.21	1.30	1.32	1.33	1.20	1.27	1.22	1.26
citric acid	1.33	1.32	1.30	1.24	1.27	1.28	1.28	1.30	1.29
formic acid	1.37	1.33	1.28	1.31	1.25	1.31	1.29	1.35	1.31
glutaric acid	1.35	1.33	1.28	1.25	1.34	1.38	1.30	1.33	1.32
acetic acid	1.33	1.35	1.28	1.29	1.32	1.30	1.37	1.34	1.32

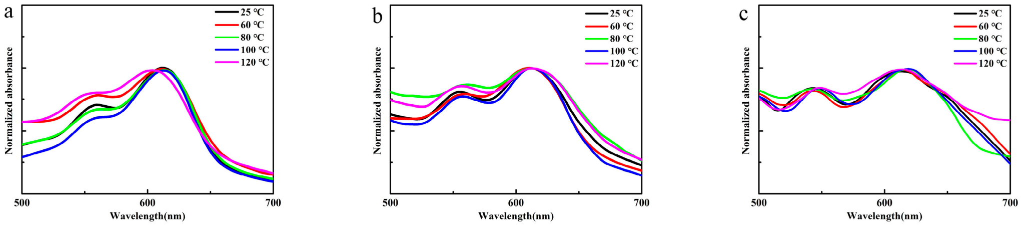

Figure S8. Normalized UV-vis absorption spectra of PBI-1 aggregates after 2 h heating. (**a**) r = 50:1 oxalic acid, (**b**) r = 300:1 formic acid, (**c**) r = 500:1 acetic acid.

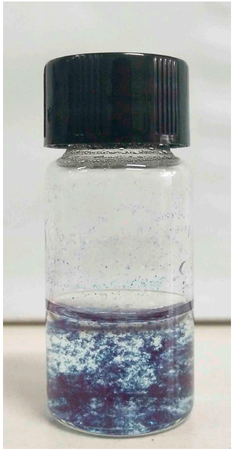

Figure S9. Digital image of PBI-1 aggregates assembled in adipic acid, r = 500:1.
